# Carrot (*Daucus carota* L.) Seed Germination Was Promoted by Hydro-Electro Hybrid Priming Through Regulating the Accumulation of Proteins Involved in Carbohydrate and Protein Metabolism

**DOI:** 10.3389/fpls.2022.824439

**Published:** 2022-02-10

**Authors:** Shuo Zhao, Hao Zou, Yingjie Jia, Xueqin Pan, Danfeng Huang

**Affiliations:** ^1^School of Agriculture and Biology, Shanghai Jiao Tong University, Shanghai, China; ^2^School of Mechanical Engineering, Institute of Refrigeration and Cryogenics, Shanghai Jiao Tong University, Shanghai, China; ^3^Shanghai Vegetable Research Institute, Shanghai, China

**Keywords:** hydro-electro hybrid priming, carrot, seed germination, TMT-based proteomic analysis, protein synthesis and degradation, carbohydrate metabolism

## Abstract

Asynchronized and non-uniform seed germination is causing obstacles to the large-scale cultivation of carrot (*Daucus carota* L.). In the present study, the combination of high voltage electrostatic field treatment (EF) with hydropriming (HYD), namely hydro-electro hybrid priming (HEHP), significantly improved all germination indicators of carrot seeds, and the promoting effect was superior to that of the HYD treatment. A tandem mass tags (TMT)-based proteomic analysis identified 4,936 proteins from the seeds, and the maximum number of differentially abundant proteins (DAPs) appeared between CK and HEHP. KEGG analysis revealed that the upregulated DAPs were mainly enriched in the pathways related to protein synthesis and degradation such as “ribosome” and “proteasome,” while the downregulated DAPs were mainly enriched in photosynthesis-related pathways. Furthermore, the maximum DAPs were annotated in carbohydrate metabolism. Some proteins identified as key enzymes of the glyoxylate cycle, the tricarboxylate cycle, glycolysis and the pentose phosphate pathway showed enhanced abundance in priming treatments. The activities of several key enzymes involved in carbohydrate metabolism were also enhanced by the priming treatments, especially the HEHP treatment. Real-time quantitative PCR (qRT-PCR) analysis revealed that the effect of priming is mainly reflected before sowing. In conclusion, the optimal effect of HEHP is to regulate the synthesis and degradation of proteins in seeds to meet the requirements of germination and initiate the utilization of seed storage reserves and respiratory metabolism. The present work expanded the understanding of the response mechanism of carrot seed germination to priming and the biological effects of high voltage electrostatic field.

## Introduction

Rapid and uniform germination is a crucial factor in determining field performance and crop yield, especially under the increasing uncertainty caused by global climatic change (Marcos-Filho, 2015; Finch-Savage and Bassel, 2016). Asynchronized and non-uniform seed germination is causing obstacles to the large-scale cultivation of carrot (*Daucus carota* L.). The mechanized precision sowing widely used in carrot cultivation also puts forward higher requirements for the germination performance of carrot seeds. Our group has developed a novel seed priming technology named hydro-electro hybrid priming (HEHP) by combining high voltage electrostatic field treatment (EF) and hydropriming (HYD). Moreover, we applied this method to carrot seeds for the first time, which expanded the model of the presowing treatment of carrot seeds.

Seed germination is essential to determine the connection between two successive plant generations, which is generally composed of three phases: rapid imbibition (phase I), metabolism reactivation (phase II) and radicle emergence (phase III) (Finch-Savage and Leubner-Metzger, 2006; Dekkers et al., 2013). Phase II is the critical phase of germination for extensively activated gene expression and metabolic activity, during which imbibed seeds either complete the germination process or remain dormant (Carrera et al., 2007; Han et al., 2017). Seed priming tends to advance some metabolic processes related to germination and avoid radicle extension by controlling the hydration state of seeds in phase II (Zhao et al., 2018; Srivastava et al., 2021). After desiccation, the seeds can still be stored for a certain period of time for commercial transportation and sales (Gallardo et al., 2001; Fabrissin et al., 2021). Seeds after priming usually show excellent germination performance by reducing the imbibition time (Espanany et al., 2016; Zhao et al., 2020; Chakma et al., 2021). As a non-chemical method, HYD is mainly carried out by soaking seeds with distilled water or controlling the water supply under optimal temperature conditions, which is environmentally safe and low cost, and the effect is remarkable (Yan, 2016; Nakao et al., 2020), but the seeds are easily infected by harmful microorganisms in the process of long-term priming (Singh et al., 2015). By applying EF treatment on the basis of HYD, HEHP achieves the goal of reducing the priming time, and this novel priming method has been successfully applied to onion seeds (Zhao et al., 2018).

At the stage of seed germination and seedling establishment, proteins are directly responsible for regulating biochemical reactions and metabolic processes in seeds (Li et al., 2019). The synthesis of specific proteins is necessary for the smooth progress of seed germination. For example, amanitine (an RNA polymerase II inhibitor) did not inhibit seed germination, while cycloheximide (a protein synthesis inhibitor) prevented the extension of the radicle (Rajjou et al., 2004; Sano et al., 2012). Studies on *Arabidopsis thaliana* have shown that more than 10,000 kinds of mRNA are stored in dry seeds (Nakabayashi et al., 2005). Within a few hours after seed imbibition, seeds can use these mRNAs and polysomal protein-synthesizing complexes appear in seeds for protein synthesis (Bewley, 1997). Meanwhile, the amino acids needed for *de novo* protein synthesis after germination are realized through the degradation of storage proteins. Various types of 20S proteasomes related to proteolysis increased abundance during germination (Rajjou et al., 2006; Wang et al., 2012). In addition, increased accumulation of proteins related to glycolysis, tricarboxylate cycle (TCA cycle) and pentose phosphate pathway were found in the germination process (Sano et al., 2012; Gu et al., 2016). The early stage of seed imbibition experienced a short anaerobic period. When the production of mitochondrial ATP is limited by hypoxia, the glycolysis pathway is dominant, while the pentose phosphate pathway is dominant after mitochondria become active (Nonogaki et al., 2010), and thus the accumulation of proteins involved in these pathways is required during early seed germination.

Proteomic techniques can effectively identify the characteristics of protein composition, which is an effective way to explore the molecular physiological basis of seed priming (Macovei et al., 2017; Marthandan et al., 2020). Numerous studies have used proteomic techniques to further uncover the molecular mechanisms of seed priming such as durum wheat (Fercha et al., 2013), maize (Araujo et al., 2021), rapeseed (Kubala et al., 2015) and alfalfa (Yacoubi et al., 2013), which focused on key proteins involved in reserve mobilization, protein metabolism, cell cycle and antioxidant protection through the analysis of the differentially abundant proteins (DAPs). However, as a representative of Apiaceae crops, the seeds of carrot come from cremocarp, so it is worth exploring whether the response of carrot seeds to priming is also related to these processes. Nevertheless, few studies have investigated the biological mechanism of seed priming on carrot seed germination and the proteome response of carrot seeds mediated by seed priming remains unknown.

In the present study, the tandem mass tags (TMT) isobaric labeling approach was applied to quantitatively analyze the proteome of carrot seeds, and through the analysis of DAPs, we focused our attention on the proteins involved in protein and carbohydrate metabolism. In conjunction with physiological determination, we explored the initiation effect of HEHP treatment on carrot seed germination and compared it with that of HYD treatment.

## Materials and Methods

### Materials and Seed Treatment Protocols

Carrot seeds (*Daucus carota* L. Naaisi) were harvested in 2019 and purchased from Shanghai Wells Seeds Co., Ltd (Shanghai, China). Seeds were stored under proper conditions (5°C and 50% relative humidity).

**FIGURE 1:**
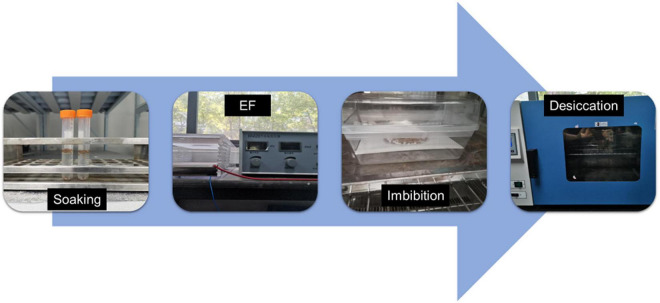
Processing protocols of the HEHP treatment. EF: high voltage electrostatic field treatment.

For the HYD and HEHP treatments, the carrot seeds were first soaked in distilled water at 20°C for 6 h. Then for HEHP, the positive and negative electrodes of a BM-201 electrostatic field generator (made in Jiangsu, China) were connected to two 10 cm × 10 cm copper plates placed 1 cm apart; the seeds were placed on the lower plate (cathode) and exposed to a 2 kV/cm EF for 90 s after absorbing the surface moisture with absorbent paper. The EF parameters were widely selected by pre-experiments. Then HYD and HEHP were imbibed in a climate chamber (QHX-300BSH-III, made in Shanghai, China) set at 22°C at 98% humidity for 48 h in the dark, and desiccated at 25°C in a drum wind dryer until the initial weight was achieved. The HEHP treatment procedures are shown in Figure 1. The control (CK) was not treated in any way. Seed germination experiments and proteomic analysis were conducted on the seed samples from the three treatments (CK, HYD and HEHP).

### Seed Germination Conditions

Fifty carrot seeds were sown in a Petri dish covered with 3 layers of sterile filter paper and moistened with 4 ml distilled water in a climate chamber (QHX-300BSH-III, made in Shanghai, China) set at 20°C and 80% relative humidity. The photoperiod was set to 14/10 h of light/dark obtained by using TL lights (TLD 30W/865 cool daylight, PHILIPS, made in Tailand) set at 40 μmol/(m^2^⋅s). Distilled water (0.5 ml) was added to the Petri dish every 24 h after sowing. A seed was considered germinated when the radicle protruded through the seed coat. The number of germinated seeds and germination percentage (%) every 24 h were recorded after sowing until fifth day after sowing. Average radicle length (RL, mm) and fresh weight per 10 randomly selected seedlings (FW, g) were determined on the fifth day. The germination index (GI) and vigor index (VI) were calculated by the following formula. A total of 18 samples (3 treatments × 6 biological replications) were used for germination test.


GI=∑Gt/Dt

VI=GI×FW

Dt is the number of days for counting the number of germinated seeds, and Gt is the number of germinated seeds counted corresponding to the number of days.

### Total Protein Extraction

The seed samples used for proteomic analysis were dry seeds with different treatments. The samples of carrot seeds (0.5 g per sample, approximately 200 seeds) were ground into a fine powder in liquid nitrogen. Then the powder was suspended in lysis buffer (8 M urea + 1% SDS, including protease inhibitor). The mixture was allowed to settle at 4°C for 30 min, and processed by ultrasound for 2 min at 0°C. After centrifugation at 12,000g at 4°C for 30 min, protein quantification was performed using BCA protein assay kit (Pierce, Thermo, United States). A total of 9 samples (3 treatments × 3 biological replications) were used for analysis.

### Protein Digestion and Tandem Mass Tags Labeling

Protein digestion was performed according to the standard procedure and the resulting peptide mixture was labeled using 10-plex TMT reagent (Thermo Fisher, Art.No.90111) according to the manufacturer’s instructions. 100 μg protein taken from each sample was mixed with 100 μL of the lysate. TCEP (10 mM) was added and then it was stored at 37°C for 60 min. Then iodoacetamide (40 mM) was added and stored in dark at room temperature for 40 min. Precooled acetone was added and precipitated at −20°C for 4 h, then centrifuged at 10,000 g for 20 min, and took the precipitate. The sample was dissolved with 50 mmol/L riethylammonium bicarbonate (TEAB), then trypsin was added according to the mass ratio of 1: 50 and enzymolysis at 37°C overnight.

Tandem Mass Tags reagent was taken out at −20°C and was restored to room temperature. Then acetonitrile was added. After vortex centrifugation, TMT reagent was added per 100 μg polypeptide and incubated at room temperature for 2 h. Hydroxylamine was added and reacted at room temperature for 15 min. The same amount of labeled product was mixed in a tube and vacuum-dried.

### High Performance Liquid Chromatography Separation

The pooled samples were fractionated by ACQUITY Ultra Performance liquid chromatography (Waters, United States) with an ACQUITY UPLC BEH C18 Column (1.7 μm, 2.1 mm × 150 mm, Waters) to increase proteomic depth. Briefly, peptides were first separated with a gradient of elution (Phase B: 5 mM ammonium hydroxide solution containing 80% acetonitrile, pH 10) over 48 min at a flow rate of 200 μl/min. A total of twenty fractions were collected according to peak shape and time, combined into ten fractions, and concentrated by vacuum centrifugation.

### LC-MS/MS Analysis

Labeled peptides were analyzed by online nanoflow liquid chromatography tandem mass spectrometry performed on a 9RKFSG2_NCS-3500R system (Thermo, United States) connected to a Q Exactive Plus quadrupole orbitrap mass spectrometer (Thermo, United States) through a nanoelectrospray ion source. The C18 chromatographic column (75 μm × 25 cm, Thermo, United States) as equilibrated with solvent A (A:2% formic acid with 0.1% formic acid) and solvent B (B: 80% ACN with 0.1% formic acid). The peptides were eluted using the gradient (0–4 min, 0–5% B; 4–66 min, 5–23% B; 66–80 min, 23–29% B; 80–89 min,29–38% B; 89–91 min, 38–48% B; 91–92 min, 48–100% B; 92–105 min, 100% B; 105–106 min, 100–0% B) at a flow rate of 300 nL/min. The Q Exactive Plus was operated in the data-dependent acquisition mode to automatically switch between full scan MS and MS/MS acquisition. The survey of full scan MS spectra (m/z 350–1,300) was acquired in the Orbitrap. Then the top 20 most intense precursor ions were selected for secondary fragmentation, and the dynamic exclusion time was 18 s.

### Protein Identification, Quantification and Bioinformatics Analysis

The raw data files were analyzed using Proteome Discoverer against the carrot database.^1^ The false discovery rate (FDR) of peptide identification during library search is set to FDR ≤ 0.01. The protein contains at least one specific peptide.

The screening criteria of differentially abundant proteins (DAPs) were as follows: *P* value < 0.05 and fold change (FC) > 1.5 were identified as upregulated DAPs, *P* value < 0.05 and FC < 0.67 were identified as downregulated DAPs. Annotation of DAPs was performed using Gene Ontology (GO)^2^,^3^ and Kyoto Encyclopedia of Genes and Genomes (KEGG).^4^ DAPs were also used for KEGG enrichment analysis.

### Determination of Key Enzyme Activities

The samples used for enzyme activity determination were conducted on the seeds (0.2 g for each sample, approximately 80 dry seeds or 50 imbibed seeds) at S0 (before sowing) and S20 (during germination, 20 h imbibed after sowing). All samples were frozen in liquid nitrogen and stored in a refrigerator at −80°C. The activity of malate synthase (MS, EC 2.3.3.9) was determined by assay kits (Michy Biomedical Technology Co., Ltd., Jiangsu, China) based on the principle that malate synthase catalyzes acetyl-CoA and glyoxylate to produce malate and CoA and transforms colorless DTNB into yellow TNB, with characteristic absorbance at 412 nm. The activity of sucrose synthase (SS, breakdown direction, EC 2.4.1.13) was determined by assay kits (Comin Biotechnology Co., Ltd., Jiangsu, China) based on the principle that sucrose synthase catalyzes the production of free fructose and UDPG from sucrose and UDP and the content of reducing sugar was determined by the 3,5-dinitrosalicylic acid method to reflect the enzyme activity. The activities of 6-phosphofructokinase (PFK, EC 2.7.1.40) and glucose-6-phosphate dehydrogenase (GPDH, EC 1.1.1.49) were determined by assay kits (Comin Biotechnology Co., Ltd., Jiangsu, China) and the enzyme activities were calculated by monitoring the rate of decrease in the absorbance value at 340 nm which is the position of the NADH absorption peak. A total of 18 samples (3 treatments × 2 sampling time points × 3 biological replications) were used for the analysis of each enzyme.

### Real-Time Quantitative PCR Analysis

The samples used for real-time quantitative PCR (qRT-PCR) analysis were also conducted on the seeds (0.2 g for each sample, approximately 80 dry seeds or 50 imbibed seeds) at S0 and S20. Total RNA was extracted from the seed samples using Plant RNA Purification Reagent according to the manufacturer’s instructions (Invitrogen, Waltham, MA, United States), and genomic DNA was removed using DNase I (TaKaRa, Shiga, Japan). The primers were designed with Primer 5.0 (Supplementary Table 1). The inner reference used the carrot gene *actin-7* (LOC108202619). Reverse transcription amplification was performed with a Goldenstar RT6 cDNA synthesis kit ver 2 (Tsingke Biotechnology Co., Ltd., Shanghai, China). qRT-PCR was performed with 2 × T5 Fast qRT-PCR Mix (SYBR Green I) (Tsingke Biotech, Beijing, China) on a CFX Connect Real-Time PCR Detection System (Bio-Rad, Hercules, CA, United States). The qRT-PCR amplification parameters were as follows: 95°C for 1 min; then 40 cycles of 95°C for 15 s, 60°C for 15 s, and 72°C for 30 min; followed by 1 cycle of 95°C for 5 s, 60°C for 1 min, and 50°C for 30 s. The relative quantitative results were calculated by the 2^–ΔΔ*Ct*^ method according to the Ct values. Three biological replications were performed for each reaction.

### Statistical Analysis

Statistical analysis of the germination indicators, enzyme activities and gene expression data were carried out based on the analysis of variance (ANOVA) test with IBM SPSS Statistics 19. Duncan’s multiple range test was used to compare the mean values in different treatments.

## Results

### Effect of Priming Treatments on Carrot Seed Germination

**FIGURE 2:**
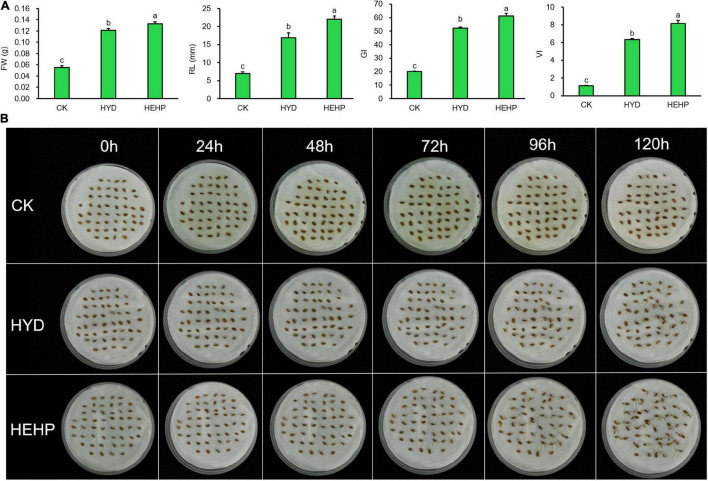
Effects of different treatments on carrot seed germination. CK, the control; HYD, hydro-priming treatment; HEHP, hydro-electro hybrid priming treatment. **(A)** Effects of different treatments on carrot seed germination characteristics. **(B)** Photograph of seed germination experiment every 24 h. Values represent the means ± SE from six biological replications. The different lowercase letters above the bars indicate significant differences in 95% probability level (*p* < 0.05, Duncan test was performed after ANOVA analysis).

As shown in Figure 2, carrot seed germination was greatly improved by priming treatments. HYD and HEHP resulted in a significant difference in the germination indicators. The FW, RL, GI and VI of HEHP were increased by 142, 218, 206, and 641% compared with those of CK, respectively (Figure 2A). Moreover, all the germination indicators of HEHP were significantly superior to those of HYD, indicating that HEHP was able to accelerate germination more adorably than single HYD. Germination was observed at 24 h after sowing in the HEHP and HYD treatments, while CK germinated at 72 h after sowing (Figure 2B).

### Identification and Quality Control of Identified Proteins

A total of 29,732 peptides and 4,936 proteins were identified in carrot seeds by MS and database searches. Among the identified proteins, proteins with molecular weights of 21–41 kDa accounted for 33%, followed by proteins with molecular weights of 41–61 and 1–21 kDa (Supplementary Figure 1A). The coverage range with the most protein quantity distribution was 20∼40, 1∼5, and 10∼20, accounting for 24, 21, and 20%, respectively (Supplementary Figure 1B). The number of proteins decreased with the increase in the number of peptides covering the protein (Supplementary Figure 1C), and the length of the peptide was generally between 7 and 13 (Supplementary Figure 1D). Principal component analysis (PCA) showed that the repeatability between the samples was high and the proteome profiles of the HEHP and HYD treatments differed from those of the control seeds, but there was a similarity between the HEHP and HYD treatments (Supplementary Figure 1E).

### Statistics of Abundant Proteins

**FIGURE 3:**
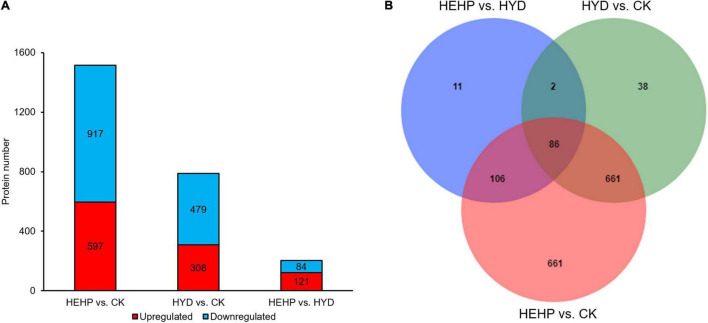
Statistics of DAPs. **(A)** The number of DAPs among the three groups. **(B)** Venn diagram showing specific and overlapping DAPs among the three groups.

As shown in Figure 3A and Supplementary Table 2, with the screening threshold of FC > 1.5 or < 0.67 and P value < 0.05, the maximum number of DAPs appeared between CK and HEHP (1514 DAPs including 597 upregulated and 917 downregulated), while the minimum number of DAPs appeared between HYD and HEHP (205 DAPs including 121 upregulated and 84 downregulated), indicating that the HEHP treatment can induce the differential expression of more proteins on the basis of the HYD treatment. Between CK and HYD, there were 787 DAPs, including 308 upregulated and 479 downregulated DAPs. The Venn diagram showed that 86 DAPs appeared simultaneously among all the groups (HEHP vs. CK, HYD vs. CK and HEHP vs. HYD), while 661, 38 and 11 DAPs specifically appeared in the HEHP vs. CK, HYD vs. CK and HEHP vs. HYD groups, respectively (Figure 3B).

### Gene Ontology and Kyoto Encyclopedia of Genes and Genomes Analysis of Abundant Proteins

**FIGURE 4:**
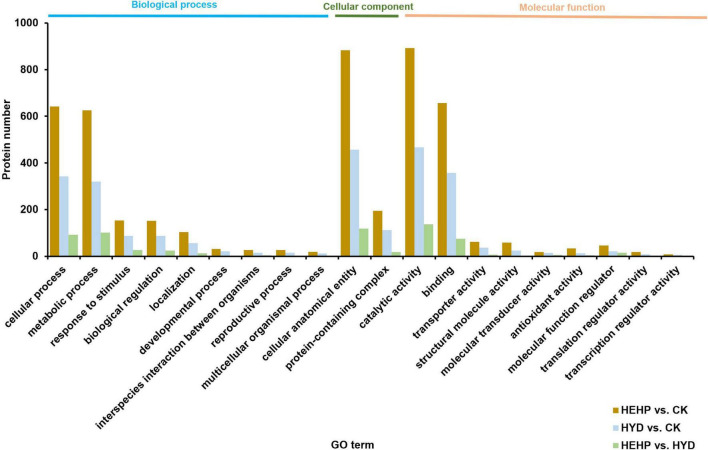
Gene ontology (GO) annotation of DAPs in HEHP vs. CK, HYD vs. CK and HEHP vs. HYD.

Annotation of DAPs was performed using GO to clarify the functional distribution (Figure 4). GO analysis showed that among all three comparisons, the GO term with the maximum DAPs was “catalytic activity” (892 DAPs in HEHP vs. CK, 467 DAPs in HYD vs. CK, 137 DAPs in HEHP vs. CK) in the molecular function category, “cellular process” (642 DAPs in HEHP vs. CK, 343 DAPs in HYD vs. CK, 92 DAPs in HEHP vs. CK) and “metabolic process” (626 DAPs in HEHP vs. CK, 320 DAPs in HYD vs. CK, 101 DAPs in HEHP vs. CK) in the biological process category, and “cellular anatomical entity” (883 DAPs in HEHP vs. CK, 457 DAPs in HYD vs. CK, 118 DAPs in HEHP vs. CK) in the cellular component category.

**FIGURE 5:**
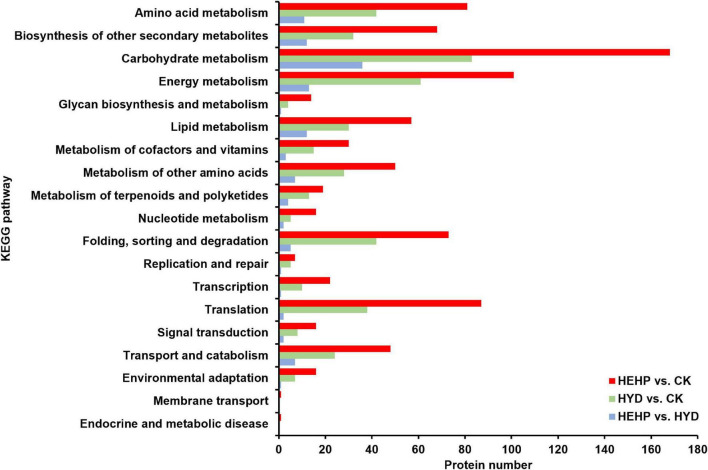
Kyoto Encyclopedia of Genes and Genomes (KEGG) annotation of DAPs in HEHP vs. CK, HYD vs. CK and HEHP vs. HYD.

KEGG pathway annotation showed that among all the three comparisons, pathways with the most DAPs were carbohydrate metabolism (168 DAPs in HEHP vs. CK, 83 DAPs in HYD vs. CK, 36 DAPs in HEHP vs. CK) and energy metabolism (101 DAPs in HEHP vs. CK, 61 DAPs in HYD vs. CK, 13 DAPs in HEHP vs. CK) (Figure 5).

To reveal the main metabolic pathways in which the DAPs participate, KEGG pathway enrichment analysis was also performed for upregulated and downregulated DAPs. The enriched KEGG pathways of each group are shown in Supplementary Table 3. For the upregulated DAPs, “ribosome” and “ribosome biogenesis in eukaryotes” were enriched in the HEHP vs. CK comparison (Supplementary Table 3A), while the “proteasome” was enriched in HYD vs. CK (Supplementary Table 3B). For the downregulated DAPs, “photosynthesis” was enriched in all three comparisons (Supplementary Tables 3D,E,F). “Photosynthesis - antenna proteins” was enriched in both the HEHP vs. CK and HEHP vs. HYD comparisons (Supplementary Tables 3D,F). In addition, “phenylpropanoid biosynthesis” was enriched in HEHP vs. CK (Supplementary Table 3D), and “carbon fixation in photosynthetic organisms” was enriched in HYD vs. CK (Supplementary Table 3E). In the HEHP vs. HYD comparisons, downregulated DAPs were also enriched in “starch and sucrose metabolism” (Supplementary Table 3F).

### Differentially Abundant Proteins Involved in the Pathways Related to Protein Synthesis and Degradation

“Ribosome” was the most enriched KEGG pathway for the upregulated DAPs in the HEHP vs. CK comparison, which had 40 upregulated DAPs in total. The proteins involved in the “ribosome” between HEHP and CK are shown in Table 1. DAPs increased in abundance that were involved in “ribosome” are mainly ribosomal proteins, including nine 40S ribosomal proteins and five 60S ribosomal proteins. The DAP with the largest FC was a ribosomal protein (A0A175YFY1). Additionally, among the four identified S-adenosylmethionine synthases (SAMs), three SAMs (A0A169WU65, A0A175YR74, A0A164YWI9) were found to have increased abundance in both the HYD and HEHP treatment, and the abundance of A0A175YR74 in the HEHP treatment was higher than that in the HYD treatment (Supplementary Table 2).

**TABLE 1:** Upregulated DAPs involved in ribosome (pathway ID: map03010) in HEHP vs. CK.

Accession	Description	FC (HEHP/CK)
A0A175YFY1	Ribosomal protein	2.873
A0A164XE28	40S ribosomal protein S12	2.422
A0A164ZZJ0	60S ribosomal protein L36	2.005
A0A165XFF0	S10_plectin domain-containing protein	1.904
A0A162AC45	S5 DRBM domain-containing protein	1.810
A0A166E4R6	60S ribosomal protein L36	1.670
A0A166AHI0	Ribosomal_L2_C domain-containing protein	1.661
A0A162BA60	40S ribosomal protein S26	1.635
A0A166D341	40S ribosomal protein S27	1.629
A0A161XAX4	40S ribosomal protein S3a	1.622
A0A165YZI7	40S ribosomal protein S8	1.611
A0A164USI6	Ribosomal_L28e domain-containing protein	1.610
A0A162AFC4	S5 DRBM domain-containing protein	1.581
A0A164ZFD9	40S ribosomal protein S3a	1.579
A0A175YRW0	40S ribosomal protein S8	1.568
A0A165YHA7	60S ribosomal protein L6	1.562
A0A166EPJ3	40S ribosomal protein SA	1.543
A0A161ZI33	Ribosomal_L18e/L15P domain-containing protein	1.537
A0A175YAJ3	60S acidic ribosomal protein P0	1.529
A0A175YPB6	60S ribosomal protein L29	1.528
A0A166ID45	40S ribosomal protein S25	1.526
A0A166FFE4	Ribosomal_S13_N domain-containing protein	1.522
A0A164WX80	Ubiquitin-like domain-containing protein	1.511
Uncharacterized proteins are not included in the table, the same below.		

The most enriched KEGG pathway of the upregulated DAPs in HYD vs. CK was the “proteasome” with nine proteins in total, which indicates that HYD may promote the advancement of protein selective degradation. As shown in Table 2, more than half of the DAPs were identified as PCI domain-containing proteins, among which the DAP with the largest FC was a PCI domain-containing protein (A0A166FNJ4). Moreover, one 26S proteasome subunit protein (A0A164WP40) was identified. The ubiquitin-proteasome pathway is the main approach of intracellular protein degradation. We also found that one ubiquitin-activating enzyme (A0A166DB90), one ubiquitin ligase (A0A164Z661), and three ubiquitin hydrolases (A0A166FN67, A0A166EMX6, A0A175YJK6) were also upregulated by priming treatments (Supplementary Table 2).

**TABLE 2:** Upregulated DAPs involved in proteasomes (pathway ID: map03050) in HYD vs. CK.

Accession	Description	FC (HYD/CK)
A0A166FNJ4	PCI domain-containing protein	1.769
A0A166DU08	PCI domain-containing protein	1.723
A0A175YJ52	MPN domain-containing protein	1.672
A0A164WP40	26S proteasome regulatory subunit RPN11	1.656
A0A175YN30	PCI domain-containing protein	1.620
A0A164Z492	AAA domain-containing protein	1.620
A0A166GAD0	PCI domain-containing protein	1.592
A0A162BAD4	PCI domain-containing protein	1.563
A0A161XSF4	AAA domain-containing protein	1.521

### Differentially Abundant Proteins Involved in Photosynthesis

Differentially abundant proteins that decreased in abundance were significantly enriched in “photosynthesis” among all three comparisons (36 DAPs in HEHP vs. CK, 23 DAPs in HYD vs. CK, 7 DAPs in HEHP vs. HYD), indicating that the expression of photosynthesis-related proteins was temporarily inhibited in the priming treatment. In the HYD vs. CK comparison, the identified DAPs involved in photosynthesis mainly contained photosystem II, photosystem I, ATP synthase subunit and ferredoxin-NADP reductase proteins (Table 3), while in the HEHP vs. CK comparison, in addition to the proteins mentioned above, the identified DAPs also contained cytochrome proteins (Table 4). In the HEHP vs. HYD comparison, the identified DAPs enriched in photosynthesis contained photosystem II, ATP synthase subunit and plastocyanin reductase proteins (Table 5).

**TABLE 3:** Downregulated DAPs involved in photosynthesis (pathway ID: map00195) in HYD vs. CK.

Accession	Description	FC (HYD/CK)
A0A166ACY1	23 kDa subunit of oxygen evolving system of photosystem II	0.371
A0A164UZK5	23 kDa subunit of oxygen evolving system of photosystem II	0.391
A0A175YCN0	ATP synthase subunit alpha	0.470
A0A175YC95	ATP synthase subunit beta	0.536
A0A165Y0P6	Plastocyanin	0.552
A0A165Y8N7	Photosystem II 10 kDa polypeptide, chloroplastic	0.560
A0A162AF45	Plastoquinol–plastocyanin reductase	0.561
A0A175YED7	Photosystem II protein D1	0.565
A0A175YDB1	Photosystem I P700 chlorophyll a apoprotein A2	0.576
A0A175YDE5	Photosystem II CP43 reaction center protein	0.578
A0A164XM95	Ferredoxin–NADP reductase, chloroplastic	0.590
A0A175YCC1	Photosystem II CP47 reaction center protein	0.622
A0A175YEF6	Photosystem I P700 chlorophyll a apoprotein A1	0.635
A0A175YCP3	Photosystem II D2 protein	0.640
A0A175YCE7	Photosystem I iron-sulfur center	0.643

**TABLE 4:** Downregulated DAPs involved in photosynthesis (pathway ID: map00195) in HEHP vs. CK.

Accession	Description	FC (HEHP/CK)
A0A164UZK5	23 kDa subunit of oxygen evolving system of photosystem II	0.270
A0A166ACY1	23 kDa subunit of oxygen evolving system of photosystem II	0.294
A0A162AF45	Plastoquinol–plastocyanin reductase	0.315
A0A165Y8N7	Photosystem II 10 kDa polypeptide, chloroplastic	0.368
A0A175YCN0	ATP synthase subunit alpha	0.385
A0A164XM95	Ferredoxin–NADP reductase, chloroplastic	0.416
A0A175YED7	Photosystem II protein D1	0.434
A0A175YDB1	Photosystem I P700 chlorophyll a apoprotein A2	0.440
A0A175YDE5	Photosystem II CP43 reaction center protein	0.446
A0A165Y0P6	Plastocyanin	0.446
A0A175YDD0	ATP synthase CF0 B subunit	0.447
A0A175YC95	ATP synthase subunit beta	0.465
A0A166A8H7	Photosystem II 10 kDa polypeptide, chloroplastic	0.494
A0A175YCE7	Photosystem I iron-sulfur center	0.496
A0A175YCI3	Cytochrome f	0.515
A0A175YCP3	Photosystem II D2 protein	0.519
A0A175YEF6	Photosystem I P700 chlorophyll a apoprotein A1	0.521
A0A175YCS2	Cytochrome b6	0.536
A0A175YCC1	Photosystem II CP47 reaction center protein	0.560
A0A161Y8Y8	Ferredoxin–NADP reductase, chloroplastic	0.574
A0A161Y432	PSI-K	0.577
A0A161ZVZ6	Ferredoxin–NADP reductase, chloroplastic	0.583
A0A164VGA4	23 kDa subunit of oxygen evolving system of photosystem II	0.594
A0A166CIQ3	PSI subunit V	0.660

**TABLE 5:** Downregulated DAPs involved in photosynthesis (pathway ID: map00195) in HEHP vs. HYD.

Accession	Description	FC (HEHP/HYD)
A0A162AF45	Plastoquinol–plastocyanin reductase	0.562
A0A175YCS2	Cytochrome b6	0.601
A0A166A8H7	Photosystem II 10 kDa polypeptide, chloroplastic	0.651
A0A165Y8N7	Photosystem II 10 kDa polypeptide, chloroplastic	0.657
A0A175YDD0	ATP synthase CF0 B subunit	0.667

### Differentially Abundant Proteins Involved in Carbohydrate Metabolism

**FIGURE 6:**
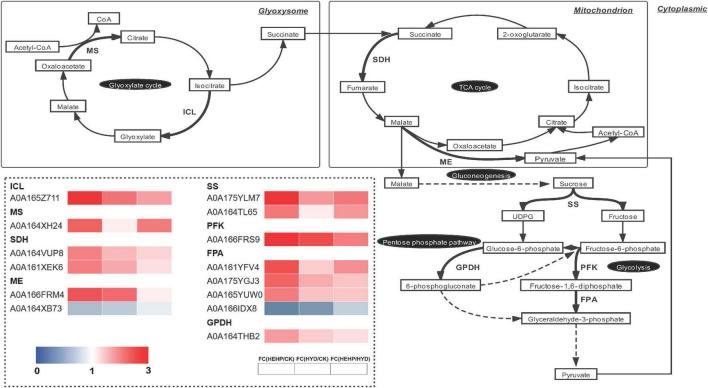
Differentially abundant proteins involved in the glyoxylate cycle and respiratory metabolism. ICL, isocitrate lyase; MS, malate synthase; SDH, succinate dehydrogenase; ME, malic enzyme; SS, sucrose synthase; PFK, 6-phosphofructokinase; FPA, fructose-bisphosphate aldolase; GPDH, glucose 6-phosphate dehydrogenase.

As shown in the analysis of KEGG pathway annotation, DAPs annotated as “carbohydrate metabolism” were the most among all three comparisons (Figure 5). The Venn analysis of these DAPs among the three comparisons suggests that almost all the DAPs in the HYD vs. CK and HEHP vs. HYD comparisons were included in the HEHP vs. CK comparison, except three DAPs in the HYD vs. CK comparison and two DAPs in the HYD vs. CK comparison (Supplementary Figure 2). This result indicates that the role of HEHP is realized by inducing more DAPs on the basis of HYD. From the DAPs annotated as “carbohydrate metabolism” (Supplementary Table 4), it is noteworthy that among the 14 DAPs encoding key enzymes of the glyoxylate cycle and respiratory metabolism, 12 DAPs increased in abundance in the priming treatments (Figure 6). One PFK (A0A166FRS9) was identified as upregulated DAP among all three comparisons. One isocitrate lyase (ICL: A0A165Z711) and one malic enzyme (ME: A0A166FRM4) were identified as upregulated DAPs in both the HEHP and HYD treatments compared with the control. Moreover, two succinate dehydrogenase subunits (SDH: A0A164VUP8 and A0A161XEK6), two SS (A0A175YLM7 and A0A164TL65), three fructose-bisphosphate aldolases (FPA: A0A161YFV4, A0A175YGJ3, A0A165YUW0), one GPDH (A0A164THB2), and one MS (A0A164XH24) were only significantly upregulated by HEHP treatment but were not upregulated by HYD treatment.

### Activities of Key Enzymes Involved in Carbohydrate Metabolism

**FIGURE 7:**
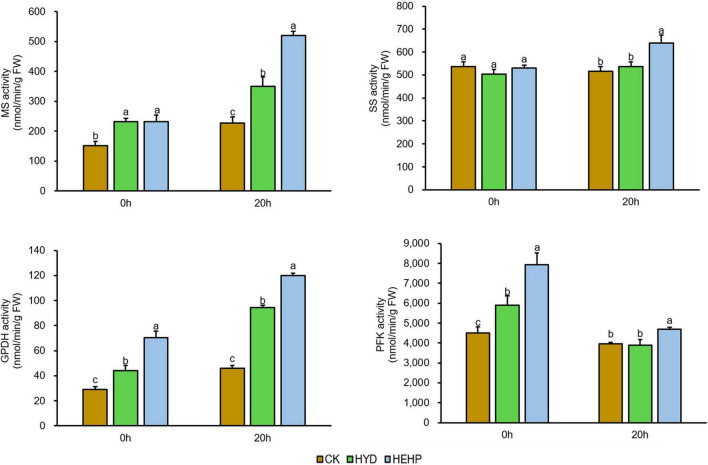
Effects of different treatments on key enzyme activities. MS, malate synthase; SS, sucrose synthase; GPDH, glucose-6-phosphatedehydrogenase; PFK, 6-phosphofructokinase. Values represent the means ± SE from six biological replications. The different lowercase letters above the bars indicate significant differences in 95% probability level (*p* < 0.05, Duncan test was performed after ANOVA analysis).

Based on the proteomic results, the activities of several key enzymes involved in carbohydrate metabolism were selected and further investigated, as shown in Figure 7. Except for SS, the activities of MS, GPDH and PFK were significantly increased by the HEHP and HYD treatments at S0, and for GPDH and PFK, the promoting effect of HEHP was significantly superior to HYD. In addition, the activities of all four enzymes were significantly increased by the HEHP treatment at S20, while for the activities of SS and PFK, there was no significant change between HYD and CK. The activities of all these enzymes in the HEHP treatment were also higher than those in the HYD treatment at S20.

### Expression Pattern of Selected Differentially Abundant Proteins at the Transcriptional Level

**FIGURE 8:**
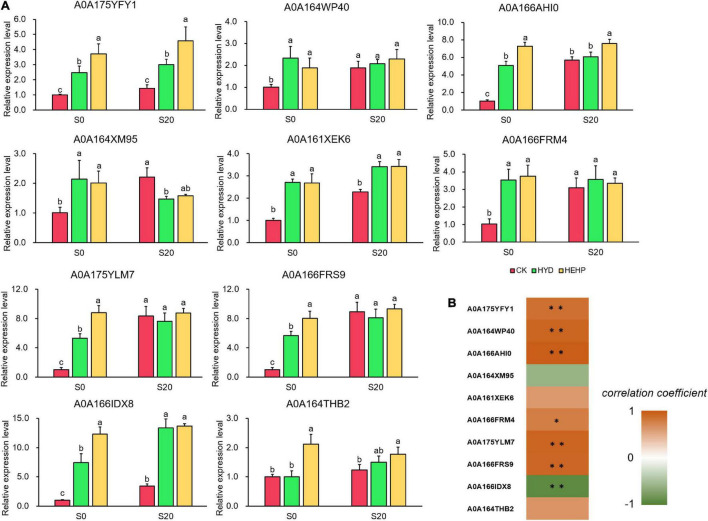
Real-time quantitative PCR (qRT-PCR) analysis of 10 selected DAPs at S0 and S20. **(A)** Expression patterns at the transcriptional level of 10 selected DAPs from qRT-PCR; **(B)** correlation analysis between the results of qRT-PCR and the proteome. Spearman correlation coefficient was used. *Means significant at the level of *p* < 0.05. ^**^Means significant at the level of *p* < 0.01. The different lowercase letters above the bars indicate significant differences in 95% probability level (*p* < 0.05, Duncan test was per-formed after ANOVA analysis).

To further explore the expression pattern of key genes encoding DAPs, ten DAPs were selected for qRT-PCR analysis at the transcriptional level from the seed samples at S0 and S20. As shown in Figure 8A, all of the selected genes were upregulated by HEHP treatment at S0, and the expression of some DAPs (A0A175YFY1, A0A166AHI0, A0A175YLM7, A0A166FRS9, A0A166IDX8, and A0A164THB2) at the transcriptional level in the HEHP treatment was significantly higher than that in the HYD treatment. The expression of A0A164THB2 at the transcriptional level was not upregulated by HYD treatment. Moreover, it was found that the expression of five genes encoding A0A164WP40, A0A164XM95, A0A166FRM4, A0A175YLM7, and A0A166FRS9 was not upregulated by the priming treatments after imbibition at S20. From the correlation analysis between the results of the expression pattern of genes and the proteomic data, five DAPs (A0A175YFY1, A0A164WP40, A0A166AHI0, A0A175YLM7, and A0A166FRS9) showed a significant positive correlation with the gene expression data while only A0A166IDX8 showed a significant negative correlation with the gene expression data (Figure 8B).

## Discussion

Germination performance is an important criterion for seed lots as it takes into consideration seed vigor through the rate, timing, and uniformity of germination (Fabrissin et al., 2021). In the present work, the germination performance of carrot seeds was greatly promoted by HEHP treatment. Numerous seed priming techniques reported recently have applied exogenous growth regulators to obtain more significant effects, such as melatonin (Yan et al., 2020), ascorbic acid (Salemi et al., 2019) and some kinds of phytohormones (Hussain et al., 2016; Huang et al., 2020; Zhao et al., 2020; Chen et al., 2021). Nevertheless, the HEHP treatment used in our study is a physical method without using any exogenous growth regulator or chemical reagent, which means that this method entirely relies on activating the vigor of the seed itself to accelerate the germination process and will not cause any chemical substance penetration or residue on the seeds, thus, it is environmentally safe. Moreover, through applying EF treatment on the basis of HYD, the effect of seed priming was more remarkable compared with using HYD alone.

### Priming Treatments Altered the Pattern of Protein Composition in Carrot Seeds

To the best of our knowledge, the present study is the first report on the response of the carrot seed proteome to seed priming by applying a TMT based proteomic technique. Both HYD and HEHP induced a large number of DAPs compared to the control seeds, while the HEHP treatment induced more DAPs than HYD, and DAPs between HEHP and HYD are mainly upregulated, which suggests that the HEHP treatment can further expand the effect of priming on the internal metabolic activities of seeds on the basis of HYD. Furthermore, compared with CK, DAPs induced by HEHP and HYD were mainly downregulated, indicating that priming treatments regulate seed germination largely by reducing the abundance of specific proteins.

### Priming Treatments Induced Protein Synthesis and Degradation

Ribosomes are composed of ribosomal proteins and rRNA, which bind to mRNAs to participate in protein synthesis when seed germination begins (Zhang et al., 2016; Bai et al., 2020). Previous studies have suggested that biologically defective mutations in ribosomes tend to lead to a low germination rate (Abbasi et al., 2010; Maekawa et al., 2018). In our study, the upregulated DAPs between HEHP and CK were most enriched in “ribosome,” and both ribosomal large subunit proteins and ribosomal small subunit proteins were found to significantly increase in abundance in HEHP, indicating that HEHP treatment plays a key role in improving the efficiency of protein synthesis. This result is consistent with previous research on cucumber (Zhang et al., 2017), rapeseed (Kubala et al., 2015) and *H. moellendorffii* (Li et al., 2019) seeds. In the present work, three SAMs were found to have increased abundance in priming treatments. Previous proteomic studies have also found that proteins related to methionine metabolism, including SAMs, increased accumulation during seed germination (Gallardo et al., 2002; Wang et al., 2012) and after priming (Fercha et al., 2013). SAMs use methionine to catalyze S-adenosylmethionine, which is involved in protein synthesis as a methyl donor (Takahashi et al., 2011). Previous study also pointed out that the recovery of seed vigor by seed priming is related to the maintenance of methionine metabolism (Yacoubi et al., 2013).

In addition to protein synthesis, selective protein degradation is also essential in the initiation process of seed germination (Muntz et al., 2001). The proteasome is a protease complex that degrades misfolded or damaged proteins, which can be ubiquitin-dependent (proteins are first labeled by ubiquitin) and directly involved in regulating the level of some key proteins (Sadanandom et al., 2012). The present results suggested that the upregulated DAPs between HYD and CK were most enriched in “proteasome,” and DAPs identified as proteasome proteins, ubiquitin-activating enzymes, ubiquitin ligases, and ubiquitin hydrolases were significantly increased in abundance in priming treatments, which implied that the ubiquitin-proteasome pathway was advanced in priming treatment. A previous study also showed that the abundance of the 26S proteasome component increased when seeds germinated under suitable conditions, and proteasome activity was high during phase I to the end of phase II of germination (Xia et al., 2018). Additionally, five PCI domain-containing proteins were identified as upregulated DAPs. The PCI domain is frequently found in the subunits of the proteasome, and is related to the regulation of protein ubiquitination and degradation (Zhu et al., 2018).

### Priming Treatments Decreased the Accumulation of Photosynthesis-Related Proteins in Carrot Seeds

In previous studies on seed germination, photosynthesis has rarely received much attention, because seed germination is an autotrophic process that mobilizes seed reserves to maintain growth, and photosynthesis does not occur (Deruyffelaere et al., 2015; Bai et al., 2020). In our study, it is noteworthy that in the HYD and HEHP treatments, we found that many DAPs involved in “photosynthesis” decreased in abundance, and the expression of some photosynthetic proteins in HEHP was even lower than that in HYD, such as A0A162AF45, A0A175YCS2, A0A166A8H7, A0A165Y8N7 and A0A175YDD0. Recent research on barley showed that photosynthesis-associated genes were downregulated during early germination (Zhu et al., 2020), which is similar to the present results. One possible explanation for this phenomenon is that due to the limited storage reserves of carrot seeds, the treatments of rapid germination (HEHP and HYD) need to concentrate all the material and energy supply in the early stage of germination to meet the requirements of germination. Therefore, the expression of photosynthesis-related proteins for heterotrophic growth is temporarily inhibited.

### Priming Treatments, Especially Hydro-Electro Hybrid Priming, Initiated Storage Reserves Utilization and Respiratory Metabolism

Dry seeds contain all the components required for germination and seedling establishment until the seedlings reach an autotrophic state (Bai et al., 2020). Proteins related to energy metabolism may show increased abundance by seed priming (Araujo et al., 2021). Carrot seeds have an endosperm with a high lipid content (mainly in the form of triacylglycerol), which is degraded to provide carbon skeleton and energy for seedling growth during germination (Ngo-Duy et al., 2009; Pandey et al., 2019). A previous study in *Arabidopsis thaliana* showed that most sucrose used for respiratory metabolism is produced by storage lipid degradation rather than other soluble sugars in seeds (Pritchard et al., 2002). The utilization of triacylglycerol (TAG) in seeds depends on the glyoxylate cycle during germination, which uses β-acetyl-CoA produced by oxidation of fatty acids is converted to gluconeogenesis and then used for energy supply during germination (Eastmond and Graham, 2001). ICL and MS are the key enzymes in the glyoxylate cycle, and both were identified as upregulated DAPs in the HEHP treatment, which suggests that HEHP has prepared key substances for the utilization of seed storage. We further determined the MS activity and found that it increased significantly by the priming treatments. Existing studies have also found the expression and activities of ICL and MS were increased during germination (Bellieny-Rabelo et al., 2016; Faraoni et al., 2019). When stored lipids are converted to sucrose after gluconeogenesis, they can be decomposed into glucose and fructose by SS and then enter glycolysis or the pentose phosphate pathway (Yu et al., 2014; Huang et al., 2021). SS is also involved in the synthesis of the cell wall, which is an essential process underlying cellular expansion during germination (Wei et al., 2015; Bellieny-Rabelo et al., 2016). Our results showed that two SSs increased in abundance by HEHP and the activity of SS was also increased by HEHP treatment during imbibition. A recent study also indicated that SS participates in the release of seed dormancy (Liu et al., 2020). Early activation of respiratory pathways was also found, which is responsible for providing energy for cell activities and the carbon skeleton of macromolecular biosynthesis during seed germination. Both PFK and FPA were identified as upregulated DAPs in the HEHP treatment, which are involved in glycolysis and PFK is one of the rate-limiting enzymes of this pathway. PFK activity was also found to be increased by HEHP treatment at both S0 and S20. It has been proposed that glycolysis-related enzymes increase in expression during early germination and the energy needs of germination seem to be met mostly by glycolysis (Yang et al., 2007; Yu et al., 2014; Xu et al., 2016). As a key enzyme of the pentose phosphate pathway, GPDH was identified as an upregulated DAP in the HEHP treatment, the activity of which was also increased by the HEHP treatment. The pentose phosphate pathway is also considered to play a key role in energy supply in the early stage of seed germination (Huang et al., 2021). Both SDH and ME participate in the TCA cycle in mitochondria, which enables the intermediate products of the TCA cycle such as malate to be completely oxidized and decomposed and can provide flexibility for the metabolism of phosphoenolpyruvate and malate (Lincoln and Eduardo, 2015). In brief, the increased abundance of these enzymes in priming treatments especially the HEHP treatment reflects that priming has induced material preparation for efficient energy supply during germination in advance. In addition, it is noteworthy that HYD did not increase the abundance of some proteins such as SDH subunits, SSs, FPAs and MA, while HEHP significantly increased the abundance of these proteins, and the results of enzyme activity determination were similar, which is another possible explanation for the more remarkable effect of HEHP than that of HYD.

### The Effect of Priming Is Mainly Reflected Before Sowing

The results of the expression pattern of the genes encoding DAPs by qRT-PCR revealed that the expression level of some selected genes was not upregulated by the priming treatments after imbibition, indicating that the effect of priming is mainly reflected in regulating gene expression before sowing. When the carrot seeds are sown and begin to imbibe, the activation effect on gene expression will gradually dissipate, which means priming has completed its mission. The abundance of one DAP (A0A166IDX8) showed significant a negative correlation with gene expression data, which is also common in previous studies and is usually attributed to temporal differences in expression or posttranslational modification (Guo et al., 2012; Li et al., 2019; Yan and Mao, 2021).

## Conclusion

**FIGURE 9:**
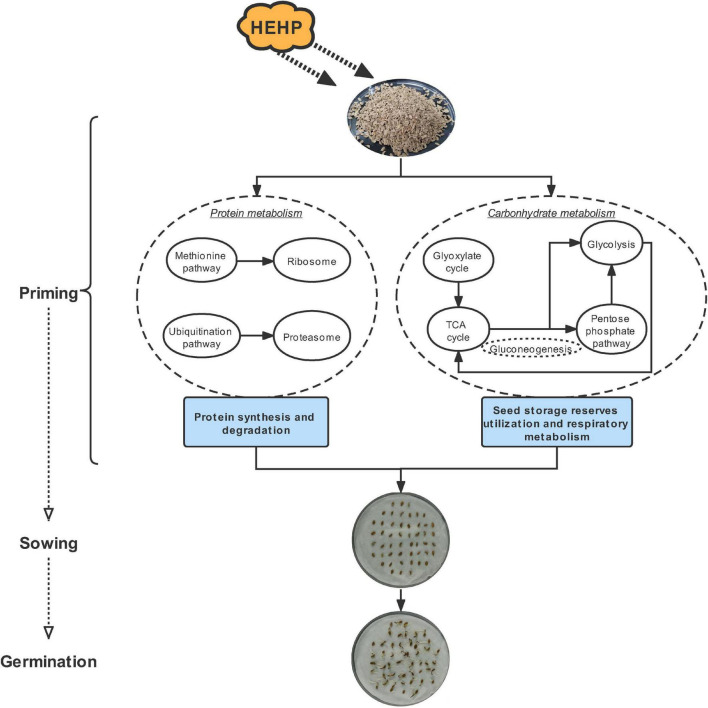
A possible regulation pathway of HEHP promoting carrot seed germination.

The seed priming treatments used in the current study substantially shortened the germination process and improved the germination performance of carrot seeds. The application of HEHP contributed to promoting the germination of carrot seeds to a greater extent than hydropriming. Proteomic analysis revealed that the priming treatments significantly altered the protein composition of carrot seeds. The upregulated DAPs were mainly enriched in the pathways related to protein synthesis and degradation, while the downregulated DAPs were mainly enriched in photosynthesis-related pathways. Furthermore, given that the maximum DAPs were annotated in carbohydrate metabolism, we focused on the critical proteins involved in the glyoxylate cycle, sucrose metabolism and glycolysis induced by the priming treatments and found that some proteins encoding key enzymes of the above pathways showed enhanced abundance in priming treatments, especially the HEHP treatment, such as ICL, MS, PFK, FPA, SDH and ME. Meanwhile, the activities of the key enzymes were also enhanced by the priming treatments, and the HEHP treatment was able to improve enzyme activities to a greater extent. The effect of priming is mainly reflected before sowing and the activation effect on gene expression will gradually dissipate after imbibition. Overall, the optimal effect of HEHP is to regulate the synthesis and decomposition of proteins in seeds to meet the requirements of germination and initiate the utilization of seed storage reserves and respiratory metabolism (Figure 9). Future research is needed to focus on the changes in metabolites and other components of the above pathways in primed seeds during germination and to keep paying attention to the inhibitory effect of seed priming on photosynthesis-related pathways.

## Data Availability Statement

The datasets presented in this study can be found in online repositories. The names of the repository/repositories and accession number(s) can be found below: ProteomeXchange via PRIDE (Project accession: PXD030133).

## Author Contributions

SZ and DH designed the experiments. SZ and HZ carried out the experiments. SZ and XP took the samples. SZ and YJ analyzed the data and prepared the tables and figures. SZ drafted the manuscript. YJ and DH improved the manuscript in English. All authors contributed to the article and approved the submitted version.

## Conflict of Interest

The authors declare that the research was conducted in the absence of any commercial or financial relationships that could be construed as a potential conflict of interest.

## Publisher’s Note

All claims expressed in this article are solely those of the authors and do not necessarily represent those of their affiliated organizations, or those of the publisher, the editors and the reviewers. Any product that may be evaluated in this article, or claim that may be made by its manufacturer, is not guaranteed or endorsed by the publisher.

## Footnotes

1
https://www.uniprot.org/proteomes/UP000077755

2
http://www.blast2go.com/b2ghome

3
http://geneontology.org/

4
http://www.genome.jp/kegg/

## Abbreviations

DAPs: differentially abundant proteins
EF: high voltage electrostatic field treatment
FDR: false discovery rate
FC: fold change
FPA: fructose-bisphosphate aldolase
FW: fresh weight
GI: germination index
GPDH: glucose-6-phosphate dehydrogenase
HEHP: hydro-electro hybrid priming
HYD: hydropriming
ICL: isocitrate lyase
ME: malic enzyme
MS: malate synthase
PFK: 6-phosphofructokinase
RL: radicle length
SAM: S-adenosylmethionine synthase
SDH: succinate dehydrogenase subunit
SS: sucrose synthase
TCA cycle: tricarboxylate cycle
TMT: tandem mass tags
VI: vigor index.

## References

[B1] Abbasi N. Kim H. B. Park N. I. Kim H. S. Kim Y. K. Park Y. I. (2010). Apum23, a nucleolar Puf domain protein, is involved in pre-ribosomal Rna processing and normal growth patterning in *Arabidopsis*. *Plant J.* 64 960–976. 10.1111/j.1365-313X.2010.04393.x 21143677

[B2] Araujo G. D. S. Lopes L. D. S. Paula-Marinho S. D. O. Mesquita R. O. Nagano C. S. Vasconcelos F. R. (2021). H_2_O_2_ priming induces proteomic responses to defense against salt stress in maize. *Plant Mol. Biol.* 106 33–48. 10.1007/s11103-021-01127-x 33594577

[B3] Bai B. van der Horst S. Cordewener J. H. G. America T. Hanson J. Bentsink L. (2020). Seed-stored mRNAs that are specifically associated to monosomes are translationally regulated during germination. *Plant Physiol.* 182 378–392. 10.1104/pp.19.00644 31527088 PMC6945870

[B4] Bellieny-Rabelo D. de Oliveira E. A. G. Ribeiro E. D. Costa E. P. Oliveira A. E. A. Venancio T. M. (2016). Transcriptome analysis uncovers key regulatory and metabolic aspects of soybean embryonic axes during germination. *Sci. Rep.* 6:36009. 10.1038/srep36009 27824062 PMC5099898

[B5] Bewley J. D. (1997). Seed germination and dormancy. *Plant Cell* 9 1055–1066. 10.1105/tpc.9.7.1055 12237375 PMC156979

[B6] Carrera E. Holman T. Medhurst A. Peer W. Schmuths H. Footitt S. (2007). Gene expression profiling reveals defined functions of the ATP-binding cassette transporter COMATOSE late in phase II of germination. *Plant Physiol.* 143 1669–1679. 10.1104/pp.107.096057 17322332 PMC1851828

[B7] Chakma S. P. Chileshe S. M. Thomas R. Krishna P. (2021). Cotton seed priming with brassinosteroid promotes germination and seedling growth. *Agron. Basel* 11:566. 10.3390/agronomy11030566

[B8] Chen H. N. Tao L. Y. Shi J. M. Han X. R. Cheng X. G. (2021). Exogenous salicylic acid signal reveals an osmotic regulatory role in priming the seed germination of *Leymus chinensis* under salt-alkali stress. *Environ. Exper. Bot.* 188:104498. 10.1016/j.envexpbot.2021.104498

[B9] Dekkers B. J. W. Pearce S. van Bolderen-Veldkamp R. P. Marshall A. Widera P. Gilbert J. (2013). Transcriptional dynamics of two seed compartments with opposing roles in *Arabidopsis* seed germination. *Plant Physiol.* 163 205–215. 10.1104/pp.113.223511 23858430 PMC3762641

[B10] Deruyffelaere C. Bouchez I. Morin H. Guillot A. Miquel M. Froissard M. (2015). Ubiquitin-mediated proteasomal degradation of oleosins is involved in oil body mobilization during post-germinative seedling growth in *Arabidopsis*. *Plant Cell Physiol.* 56 1374–1387. 10.1093/pcp/pcv056 25907570

[B11] Eastmond P. J. Graham I. A. (2001). Re-examining the role of the glyoxylate cycle in oilseeds. *Trends Plant Sci.* 6 72–77. 10.1016/s1360-1385(00)01835-5 11173291

[B12] Espanany A. Fallah S. Tadayyon A. (2016). Seed priming improves seed germination and reduces oxidative stress in black cumin (*Nigella sativa*) in presence of cadmium. *Industr. Crops Prod.* 79 195–204. 10.1016/j.indcrop.2015.11.016

[B13] Fabrissin I. Sano N. Seo M. North H. M. (2021). Ageing beautifully: can the benefits of seed priming be separated from a reduced lifespan trade-off? *J. Exper. Bot.* 72 2312–2333. 10.1093/jxb/erab004 33512455

[B14] Faraoni P. Sereni E. Gnerucci A. Cialdai F. Monici M. Ranaldi F. (2019). Glyoxylate cycle activity in *Pinus pinea* seeds during germination in altered gravity conditions. *Plant Physiol. Biochem.* 139 389–394. 10.1016/j.plaphy.2019.03.042 30959447

[B15] Fercha A. Capriotti A. L. Caruso G. Cavaliere C. Gherroucha H. Samperi R. (2013). Gel-free proteomics reveal potential biomarkers of priming-induced salt tolerance in durum wheat. *J. Proteom.* 91 486–499. 10.1016/j.jprot.2013.08.010 23973468

[B16] Finch-Savage W. E. Bassel G. W. (2016). Seed vigour and crop establishment: extending performance beyond adaptation. *J. Exper. Bot.* 67 567–591. 10.1093/jxb/erv490 26585226

[B17] Finch-Savage W. E. Leubner-Metzger G. (2006). Seed dormancy and the control of germination. *New Phytol.* 171 501–523. 10.1111/j.1469-8137.2006.01787.x 16866955

[B18] Gallardo K. Job C. Groot S. Puype M. Demol H. Job V. D. (2002). Proteomics of *Arabidopsis* seed germination. A comparative study of wild-type and gibberellin-deficient seeds. *Plant Physiol.* 129 823–837. 10.2307/4280508 12068122 PMC161704

[B19] Gallardo K. Job C. Groot S. P. C. Puype M. Demol H. Vandekerckhove J. (2001). Proteomic analysis of *Arabidopsis* seed germination and priming. *Plant Physiol.* 126 835–848. 10.1104/pp.126.2.835 11402211 PMC111173

[B20] Gu J. W. Chao H. B. Gan L. Guo L. X. Zhang K. Li Y. H. (2016). Proteomic dissection of seed germination and seedling establishment in *Brassica napus*. *Front. Plant Sci.* 7:1482. 10.3389/fpls.2016.01482 27822216 PMC5075573

[B21] Guo G. Lv D. Yan X. Subburaj S. Ge P. Li X. (2012). Proteome characterization of developing grains in bread wheat cultivars (*Triticum aestivum* L.). *BMC Plant Biol.* 12:147. 10.1186/1471-2229-12-147 22900893 PMC3480910

[B22] Han C. Zhen S. Zhu G. Bian Y. Yan Y. (2017). Comparative metabolome analysis of wheat embryo and endosperm reveals the dynamic changes of metabolites during seed germination. *Plant Physiol. Biochem.* 115 320–327. 10.1016/j.plaphy.2017.04.013 28415032

[B23] Huang L. P. Zhang L. Zeng R. E. Wang X. Y. Zhang H. J. Wang L. D. (2020). Brassinosteroid priming improves peanut drought tolerance via eliminating inhibition on genes in photosynthesis and hormone signaling. *Genes* 11:919. 10.3390/genes11080919 32796553 PMC7465412

[B24] Huang X. L. Tian T. Chen J. Z. Wang D. Tong B. L. Liu J. M. (2021). Transcriptome analysis of Cinnamomum migao seed germination in medicinal plants of Southwest China. *BMC Plant Biol.* 21:270. 10.1186/s12870-021-03020-7 34116632 PMC8194011

[B25] Hussain S. Khan F. Hussain H. A. Nie L. (2016). Physiological and biochemical mechanisms of seed priming-induced chilling tolerance in rice cultivars. *Front. Plant Sci.* 7:116. 10.3389/fpls.2016.00116 26904078 PMC4746480

[B26] Kubala S. Garnczarska M. Wojtyla L. Clippe A. Kosmala A. Zmienko A. (2015). Deciphering priming-induced improvement of rapeseed (*Brassica napus* L.) germination through an integrated transcriptomic and proteomic approach. *Plant Sci.* 231 94–113. 10.1016/j.plantsci.2014.11.008 25575995

[B27] Li F. H. Yu P. Song C. H. Wu J. J. Tian Y. Wu X. F. (2019). Differential protein analysis of *Heracleum moellendorffii* Hance seeds during stratification. *Plant Physiol. Biochem.* 145 10–20. 10.1016/j.plaphy.2019.10.002 31665663

[B28] Lincoln T. Eduardo Z. (2015). *Plant Physiology*. Beijing: Science Press.

[B29] Liu X. Huang X. Kong X. X. Zhang J. Wang J. Z. Yang M. L. (2020). Sucrose synthase is involved in the carbohydrate metabolism-based regulation of seed dormancy release in *Pyrus calleryana* Decne. *J. Hortic. Sci. Biotechnol.* 95 590–599. 10.1080/14620316.2020.1740612

[B30] Macovei A. Pagano A. Leonetti P. Carbonera D. Balestrazzi A. Araujo S. S. (2017). Systems biology and genome-wide approaches to unveil the molecular players involved in the pre-germinative metabolism: implications on seed technology traits. *Plant Cell Rep.* 36 669–688. 10.1007/s00299-016-2060-5 27730302

[B31] Maekawa S. Ishida T. Yanagisawa S. (2018). Reduced expression of APUM24, encoding a novel rRNA processing factor, induces sugar-dependent nucleolar stress and altered sugar responses in *Arabidopsis thaliana*. *Plant Cell* 30 209–227. 10.1105/tpc.17.00778 29242314 PMC5810573

[B32] Marcos-Filho J. (2015). Seed vigor testing: an overview of the past, present and future perspective. *Sci. Agric.* 72 363–374. 10.1590/0103-9016-2015-0007

[B33] Marthandan V. Geetha R. Kumutha K. Renganathan V. G. Karthikeyan A. Ramalingam J. (2020). Seed priming: a feasible strategy to enhance drought tolerance in crop plants. *Intern. J. Mol. Sci.* 21:8258. 10.3390/ijms21218258 33158156 PMC7662356

[B34] Muntz K. Belozersky M. A. Dunaevsky Y. E. Schlereth A. Tiedemann J. (2001). Stored proteinases and the initiation of storage protein mobilization in seeds during germination and seedling growth. *J. Exper. Bot.* 52 1741–1752. 10.1093/jexbot/52.362.1741 11520862

[B35] Nakabayashi K. Okamoto M. Koshiba T. Kamiya Y. Nambara E. (2005). Genome-wide profiling of stored mRNA in *Arabidopsis thaliana* seed germination: epigenetic and genetic regulation of transcription in seed. *Plant J.* 41 697–709. 10.1111/j.1365-313X.2005.02337.x 15703057

[B36] Nakao Y. Sone C. Sakagami J. I. (2020). Genetic diversity of hydro priming effects on rice seed emergence and subsequent growth under different moisture conditions. *Genes* 11:13. 10.3390/genes11090994 32854382 PMC7563639

[B37] Ngo-Duy C.-C. Destaillats F. Keskitalo M. Arul J. Angers P. (2009). Triacylglycerols of Apiaceae seed oils: composition and regiodistribution of fatty acids. *Eur. J. Lipid Sci. Technol.* 111 164–169. 10.1002/ejlt.200800178

[B38] Nonogaki H. Bassel G. W. Bewley J. D. (2010). Germination-still a mystery. *Plant Sci.* 179 574–581. 10.1016/j.plantsci.2010.02.010

[B39] Pandey S. Kumari A. Shree M. Kumar V. Singh P. Bharadwaj C. (2019). Nitric oxide accelerates germination via the regulation of respiration in chickpea. *J. Exper. Bot.* 70 4539–4555. 10.1093/jxb/erz185 31162578 PMC6735774

[B40] Pritchard S. L. Charlton W. L. Baker A. Graham I. A. (2002). Germination and storage reserve mobilization are regulated independently in *Arabidopsis*. *Plant J.* 31 639–647. 10.1046/j.1365-313X.2002.01376.x 12207653

[B41] Rajjou L. Belghazi M. Huguet R. Robin C. Moreau A. Job C. (2006). Proteomic investigation of the effect of salicylic acid on *Arabidopsis* seed germination and establishment of early defense mechanisms. *Plant Physiol.* 141 910–923. 10.1104/pp.106.082057 16679420 PMC1489900

[B42] Rajjou L. Gallardo K. Debeaujon I. Vandekerckhove J. Job C. Job D. (2004). The effect of alpha-amanitin on the *Arabidopsis* seed proteome highlights the distinct roles of stored and neosynthesized mRNAs during germination. *Plant Physiol.* 134 1598–1613. 10.1104/pp.103.036293 15047896 PMC419834

[B43] Sadanandom A. Bailey M. Ewan R. Lee J. Nelis S. (2012). The ubiquitin-proteasome system: central modifier of plant signalling. *New Phytol.* 196 13–28. 10.1111/j.1469-8137.2012.04266.x 22897362

[B44] Salemi F. Esfahani M. N. Lam-Son Phan T. (2019). Mechanistic insights into enhanced tolerance of early growth of alfalfa (*Medicago sativa* L.) under low water potential by seed-priming with ascorbic acid or polyethylene glycol solution. *Industr. Crops Prod.* 137 436–445. 10.1016/j.indcrop.2019.05.049

[B45] Sano N. Permana H. Kumada R. Shinozaki Y. Tanabata T. Yamada T. (2012). Proteomic analysis of embryonic proteins synthesized from long-lived mRNAs during germination of rice seeds. *Plant Cell Physiol.* 53 687–698. 10.1093/pcp/pcs024 22383627

[B46] Singh H. Jassal R. K. Kang J. S. Sandhu S. S. Grewal K. (2015). Seed priming techniques in field crops -A review. *Agric. Rev.* 36 251–264. 10.18805/ag.v36i4.6662

[B47] Srivastava A. K. Kumar J. S. Suprasanna P. (2021). Seed ‘primeomics’: plants memorize their germination under stress. *Biol. Rev.* 96 1723–1743. 10.1111/brv.12722 33961327

[B48] Takahashi H. Kopriva S. Giordano M. Saito K. Hell R. (2011). Sulfur assimilation in photosynthetic organisms: molecular functions and regulations of transporters and assimilatory enzymes. *Annu. Rev. Plant Biol.* 62 157–184. 10.1146/annurev-arplant-042110-103921 21370978

[B49] Wang W. Q. Mller I. M. Song S. Q. (2012). Proteomic analysis of embryonic axis of *Pisum sativum* seeds during germination and identification of proteins associated with loss of desiccation tolerance. *J. Proteom.* 77 68–86. 10.1016/j.jprot.2012.07.005 22796356

[B50] Wei Z. Qu Z. Zhang L. Zhao S. Bi Z. Ji X. (2015). Overexpression of poplar xylem sucrose synthase in tobacco leads to a thickened cell wall and increased height. *PLoS One* 10:e0120669. 10.1371/journal.pone.0120669 25807295 PMC4373717

[B51] Xia Q. Maharajah P. Cueff G. Rajjou L. Prodhomme D. Gibon Y. (2018). Integrating proteomics and enzymatic profiling to decipher seed metabolism affected by temperature in seed dormancy and germination. *Plant Sci.* 269 118–125. 10.1016/j.plantsci.2018.01.014 29606208

[B52] Xu H. H. Liu S. J. Song S. H. Wang R. X. Wang W. Q. Song S. Q. (2016). Proteomics analysis reveals distinct involvement of embryo and endosperm proteins during seed germination in dormant and non-dormant rice seeds. *Plant Physiol. Biochem.* 103 219–242. 10.1016/j.plaphy.2016.03.007 27035683

[B53] Yacoubi R. Job C. Belghazi M. Chaibi W. Job D. (2013). Proteomic analysis of the enhancement of seed vigour in osmoprimed alfalfa seeds germinated under salinity stress. *Seed Sci. Res.* 23 99–110. 10.1017/s0960258513000093

[B54] Yan H. Jia S. Mao P. (2020). Melatonin priming alleviates aging-induced germination inhibition by regulating beta-oxidation, protein translation, and antioxidant metabolism in oat (*Avena sativa* L.) seeds. *Intern. J. Mol. Sci.* 21:1898. 10.3390/ijms21051898 32164355 PMC7084597

[B55] Yan H. Mao P. (2021). Comparative time-course physiological responses and proteomic analysis of melatonin priming on promoting germination in aged oat (*Avena sativa* L.) seeds. *Intern. J. Mol. Sci.* 22:811. 10.3390/ijms22020811 33467472 PMC7830126

[B56] Yan M. (2016). Hydro-priming increases seed germination and early seedling growth in two cultivars of Napa cabbage (*Brassica rapa* subsp pekinensis) grown under salt stress. *J. Hortic. Sci. Biotechnol.* 91 421–426. 10.1080/14620316.2016.1162031

[B57] Yang P. F. Li X. J. Wang X. Q. Chen H. Chen F. Shen S. H. (2007). Proteomic analysis of rice (*Oryza sativa*) seeds during germination. *Proteomics* 7 3358–3368. 10.1002/pmic.200700207 17849412

[B58] Yu Y. L. Guo G. F. Lv D. W. Hu Y. K. Li J. R. Li X. H. (2014). Transcriptome analysis during seed germination of elite Chinese bread wheat cultivar Jimai 20. *BMC Plant Biol.* 14:20. 10.1186/1471-2229-14-20 24410729 PMC3923396

[B59] Zhang G. L. Zhu Y. Fu W. D. Wang P. Zhang R. H. Zhang Y. L. (2016). iTRAQ protein profile differential analysis of dormant and germinated grassbur twin seeds reveals that ribosomal synthesis and carbohydrate metabolism promote germination possibly through the PI3K pathway. *Plant Cell Physiol.* 57 1244–1256. 10.1093/pcp/pcw074 27296714

[B60] Zhang N. Zhang H. J. Sun Q. Q. Cao Y. Y. Li X. S. Zhao B. (2017). Proteomic analysis reveals a role of melatonin in promoting cucumber seed germination under high salinity by regulating energy production. *Sci. Rep.* 7:503. 10.1038/s41598-017-00566-1 28356562 PMC5428666

[B61] Zhao T. Deng X. Xiao Q. Han Y. Zhu S. Chen J. (2020). IAA priming improves the germination and seedling growth in cotton (*Gossypium hirsutum* L.) via regulating the endogenous phytohormones and enhancing the sucrose metabolism. *Industr. Crops Prod.* 155:112788. 10.1016/j.indcrop.2020.112788

[B62] Zhao Y. Hu M. Gao Z. Chen X. Huang D. (2018). Biological mechanisms of a novel hydro-electro hybrid priming recovers potential vigor of onion seeds. *Environ. Exper. Bot.* 150 260–271. 10.1016/j.envexpbot.2018.04.002

[B63] Zhu Y. Lei Q. Li D. Zhang Y. Jiang X. G. Hu Z. H. (2018). Proteomic and biochemical analyses reveal a novel mechanism for promoting protein Ubiquitination and degradation by UFBP1, a key component of Ufmylation. *J. Proteome Res.* 17 1509–1520. 10.1021/acs.jproteome.7b00843 29533670

[B64] Zhu Y. Q. Berkowitz O. Selinski J. Hartmann A. Narsai R. Wang Y. (2020). Conserved and opposite transcriptome patterns during germination in *Hordeum vulgare* and *Arabidopsis thaliana*. *Intern. J. Mol. Sci.* 21:7404. 10.3390/ijms21197404 33036486 PMC7584043

