# Supplementary material for: Carrot (Daucus carota L.) Seed Germination Was Promoted by Hydro-Electro Hybrid Priming Through Regulating the Accumulation of Proteins Involved in Carbohydrate and Protein Metabolism
Source: Front Plant Sci. 2022 Feb 10;13:824439. doi: 10.3389/fpls.2022.824439 (PMC8868939; doi:10.3389/fpls.2022.824439)
Supplement: Supplementary file 5 [file Data_Sheet_1.docx]

**
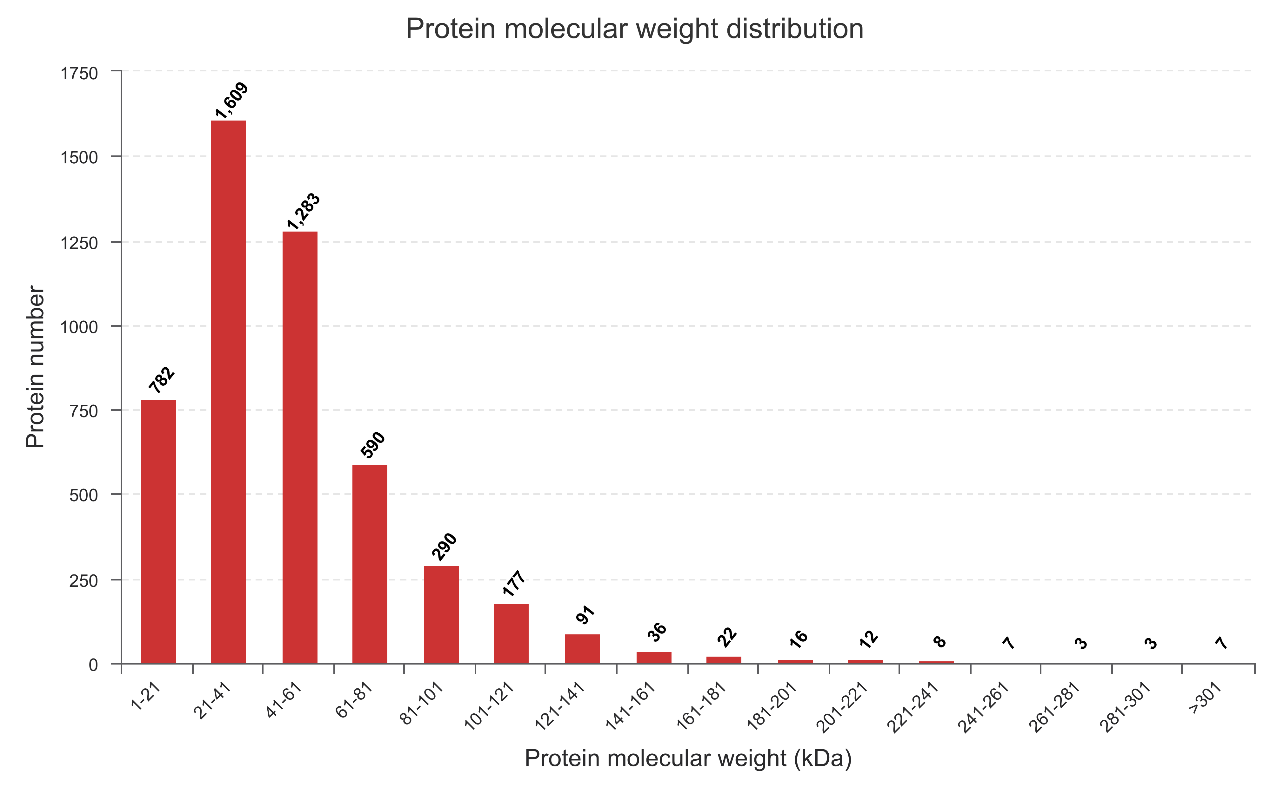
** **Supplementary Figure 1A.** Molecular weight distribution of identified proteins.

**
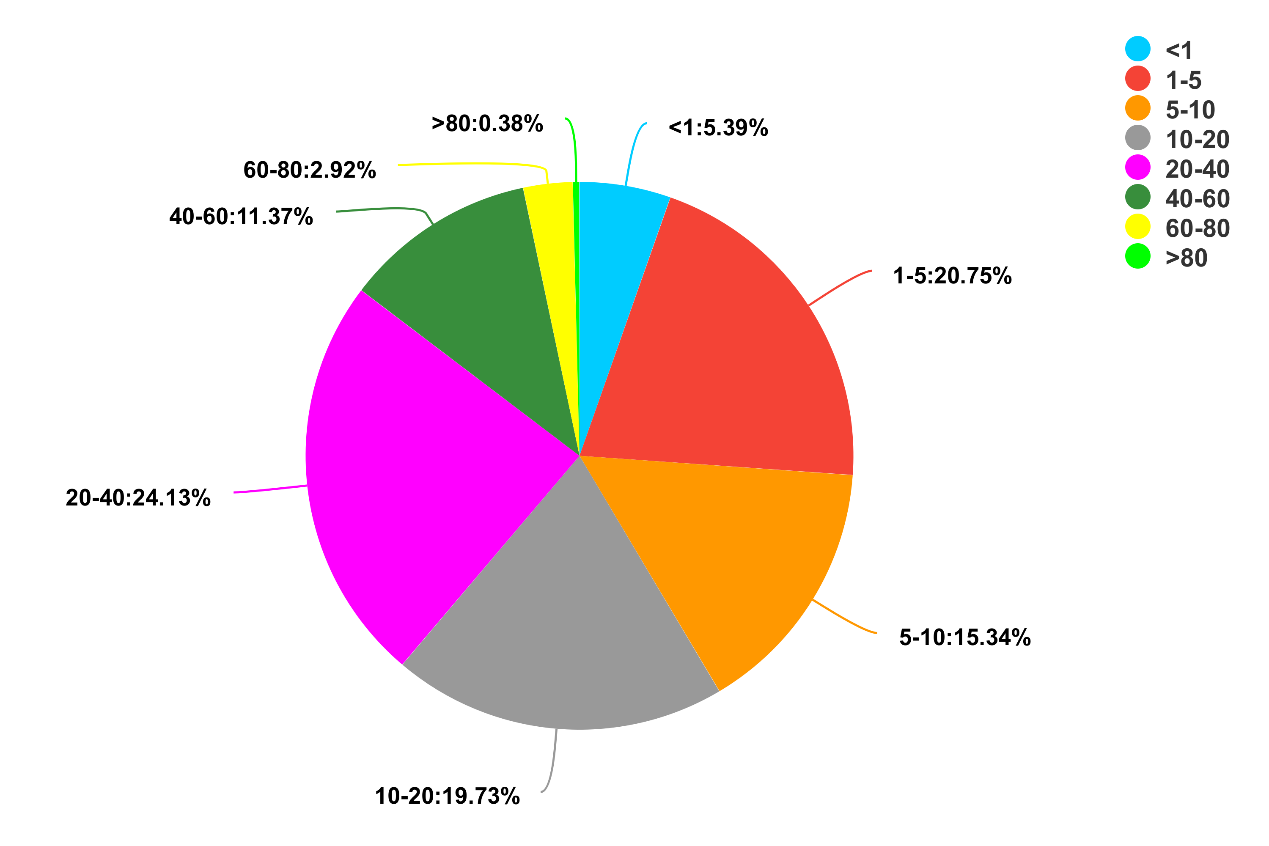
** **Supplementary Figure 1B.** Distribution of identified proteins’ sequences coverage.

**
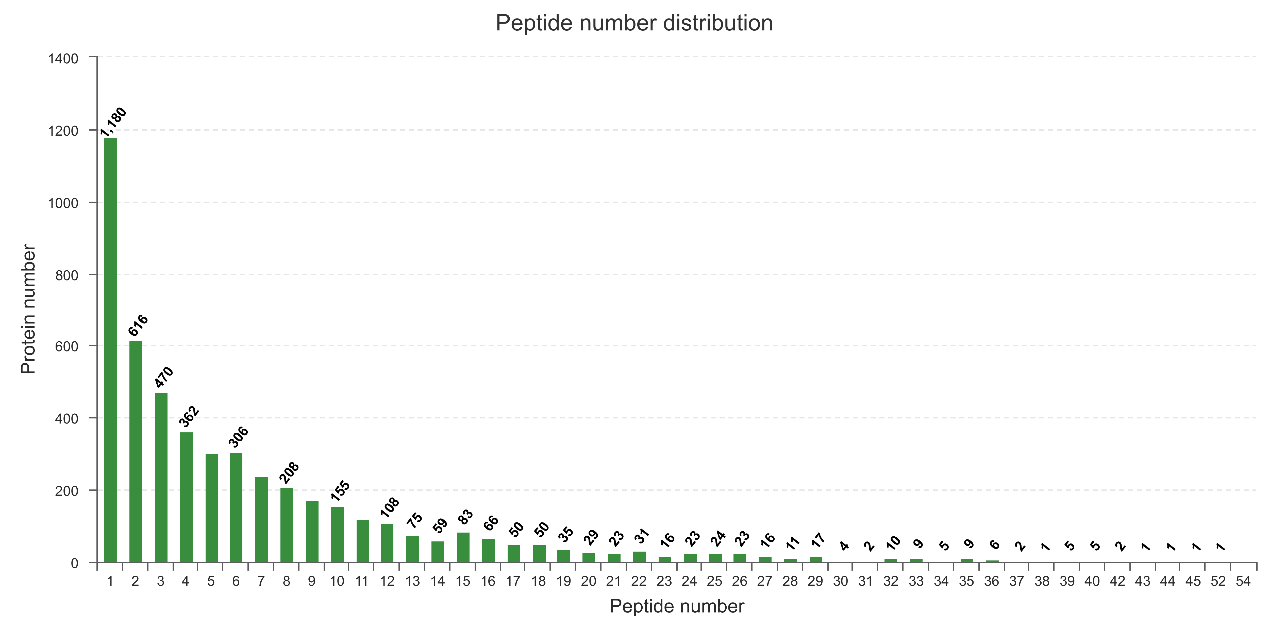
Supplementary Figure 1C.** Number distribution of identified proteins.

**
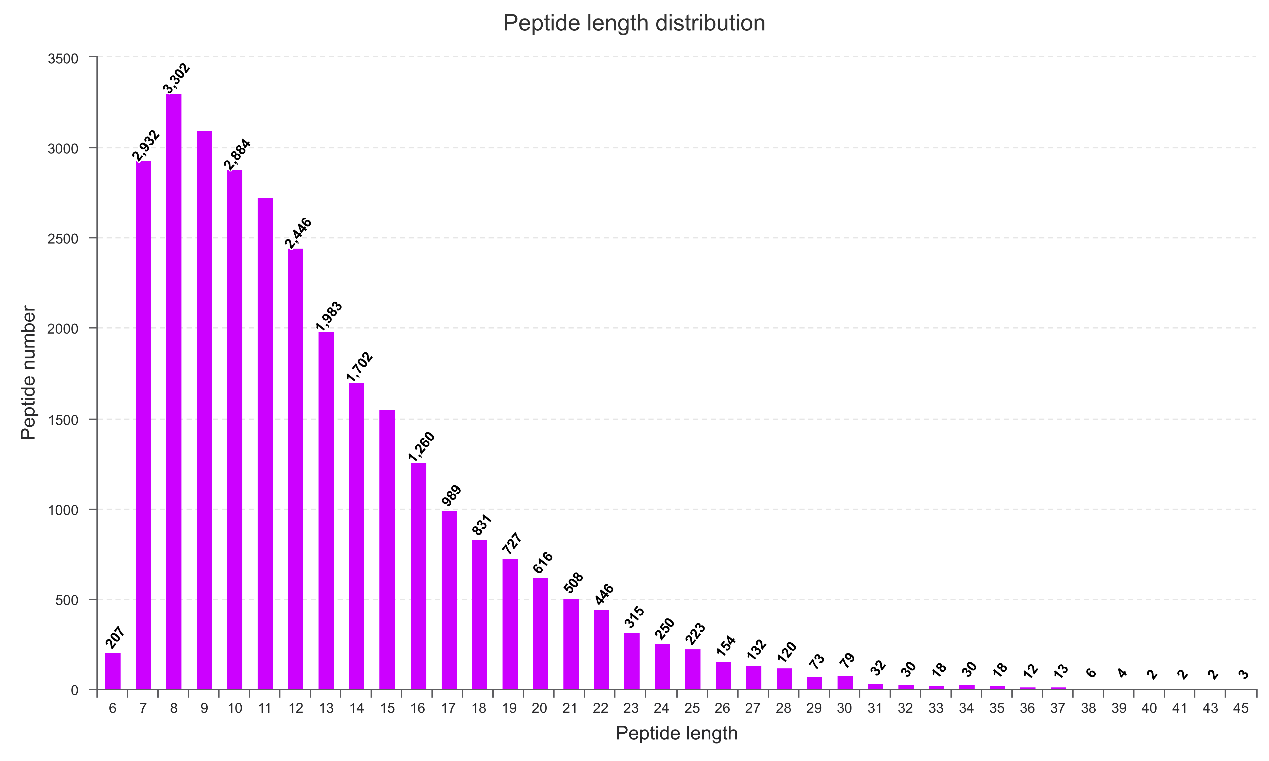
Supplementary Figure 1D.** Length distribution of peptide.

**Supplementary Figure 1E.** Principal components analysis (PCA) of proteome samples
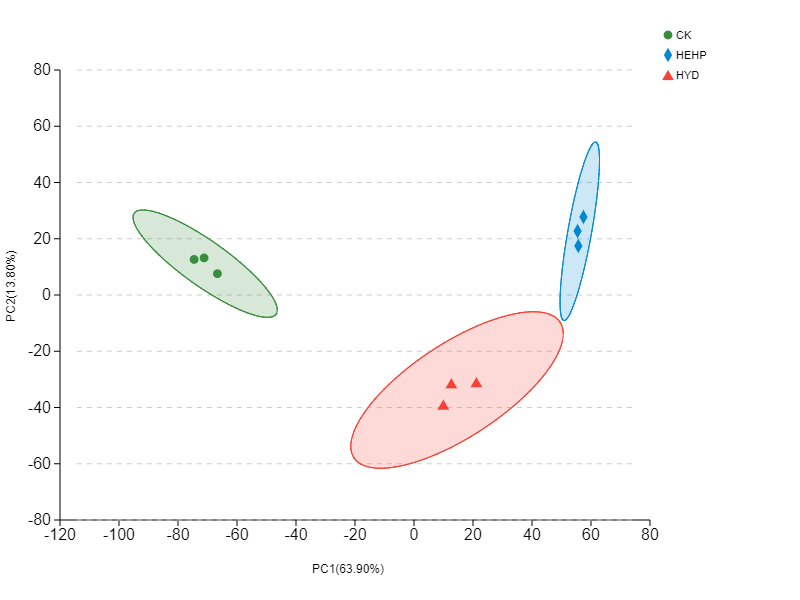
.
