# Supplementary material for: Carrot (Daucus carota L.) Seed Germination Was Promoted by Hydro-Electro Hybrid Priming Through Regulating the Accumulation of Proteins Involved in Carbohydrate and Protein Metabolism
Source: Front Plant Sci. 2022 Feb 10;13:824439. doi: 10.3389/fpls.2022.824439 (PMC8868939; doi:10.3389/fpls.2022.824439)
Supplement: Supplementary file 6 [file Data_Sheet_2.docx]

**
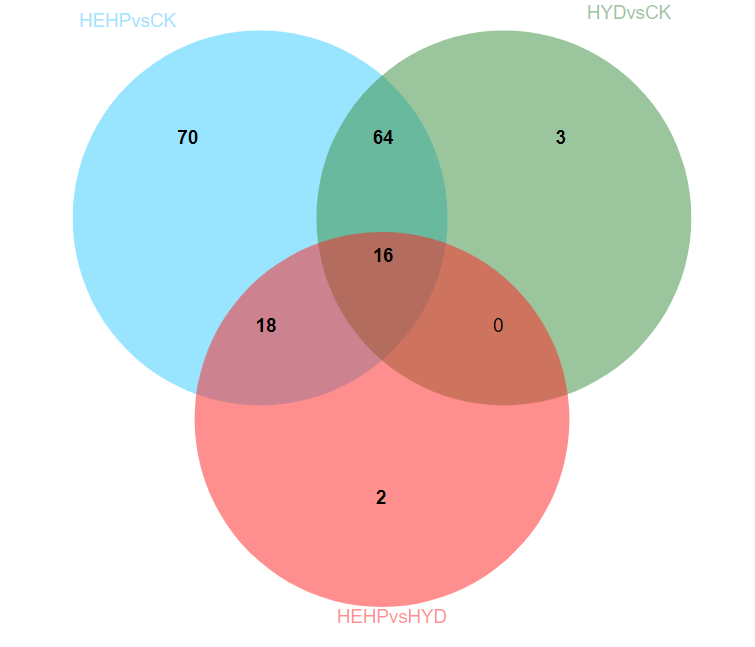
** **Supplementary Figure 2.** Venn diagram showing specific and overlapping DAPs involved in carbohydrate metabolism among the three groups.
